# How to get shortlisted for medical jobs

**DOI:** 10.1097/IJ9.0000000000000016

**Published:** 2017-06-08

**Authors:** Buket Gundogan, Kiron Koshy, Katharine Whitehurst, Alex Fowler, Riaz Agha

**Affiliations:** a University College London Medical School; b Brighton and Sussex University Hospitals; c Royal Devon and Exeter Hospital; d Guy's and St Thomas' NHS Foundation Trust, London, UK

**Keywords:** Shortlisting, Medical shortlisting, Application process, Application, Career progression, Core surgical training, Core medical training, Academic foundation program, Speciality training, Consultant

## Abstract

Progressing up the medical career ladder is a tough business. Most medical and surgical applications center the requirement for a holistic, balanced individual. As a result, there are usually specific marking criteria in predefined sections. This article provides a guide into what employers are looking for and how best to build your portfolio in these areas.

Progressing up the medical career ladder is a tough business. Many candidates find themselves waiting years before their applications are considered strong enough to be invited to interview, let alone be awarded a job. The candidates who are most likely to be successful in getting such jobs are those that knew what they had to do well ahead of time (and did those things too!).

There is no such thing as starting too early—most ventures can be started while at medical school!

## What are they looking for?

In short—they are looking for many things, the reason being—you have to distinguish yourself. Passing your medical degree, getting an intercalated degree, and good references won’t distinguish you significantly from the “crowd.”

Many application processes (eg, specialist training) have a specific marking criteria. These are explicit points-based systems, whereby if you reach the threshold number of points, then you will be shortlisted for interview. The points are based on different domains including research, teaching, and evidence of leadership. Many of these sections are also point-capped, so for example if you can only score 4 points for audits, spending time to complete 6 will not help your application. Knowing this is critical, so you can ensure you’re focusing efforts effectively!

If you haven’t yet picked a specialty, there are many things that you can do that will apply to all applications. So, what can you do to increase your chances?

## Publish scientific papers

The phrase “publish or perish” was coined due to the intense pressure felt by individuals to get scientific papers published, or “perish” in career terms. Therefore, make a special effort to publish work that you’ve carried out from a past Student Selected Component; intercalated bachelor of science (BSc) or another project of your own initiative. Any of your publications should, ideally, be published in journals that are indexed in PubMed, as points aren’t always provided for publications without a PubMed identification number (PMID).

However, don’t panic if your research gets an absolute rejection by such journals (ie, without the opportunity for a revision). If you can’t improve your paper any further, it is still worth trying to get it published in a nonindexed journal—they don’t look as impressive, but can still be put on your curriculum vitae (CV), and ensure that your efforts haven’t been wasted. Publishing letters and case reports are an easier way of building your confidence and experience. Start submitting research during your earlier years of medical school to get through this learning curve. Always pay attention to the comments you get back from editors; respect them and learn from them.

### Top tip

Start your research portfolio early, and don’t be put off by rejection. Ensure you update your CV regularly and keep it organized—this will save you a lot of time and avoid the last minute rush before applying for jobs.

## Audit and quality improvement projects

This is where you compare the observed clinical practice and results (which you objectively record) with the reference best practice guidelines developed for that procedure.

For example, a common audit is to look at how many patients on your ward have been risk assessed for venous thromboembolisms and compare with the national guideline—that every admitted patient should be venous thromboembolisms risk assessed. If the levels of risk assessment are lower than the given standard, then you may provide an intervention to improve this, such as incorporating the risk assessment into an admission proforma. Finally, you would reassess the prevalence of risk assessment to see if implementation of the changes you recommended made any difference. This is called “closing the audit loop.” Try to close the loop on every audit you carry out, as it often provides more points on applications.

Audit help to improve adherence to a “best” reference standard of practice. At the end of the audit you make recommendations on what can be done to improve adherence to the standard.

### Top tips

Try and get audits published and aim for at least 3 reaudit cycles.

## Further qualifications

These show that you have taken time to further your interests, and make you stand out from others. As a medical student, doing an intercalated degree or completing an MBPhD are ways to gain qualifications. As a junior doctor, there are many Masters Courses, MPhils, and PhD programs available.

### Top tip

The more advanced the qualification, the better!

## Presentations

Presenting research work at Local, Regional, National, and International Conferences can be a good boost to a CV and show that you are taking part in the wider scientific and clinical community. They also help to raise your profile and allow you to meet future research and clinical supervisors. When choosing which conference to submit your work to for presentation, either as a poster or oral (oral presentations are worth more), look to see which ones will abstract your work into a supplementary issue of a journal. An abstract goes onto your publication record and helps to add further clout to your CV, although this won’t count for points in the shortlisting process (you will still have the presentation and can publish the full paper in another journal if you wish, so consider this a bonus).

### Top tips

Aim for an oral presentation at an international conference. Keep any evidence of your presentation for your portfolio, for example, the conference program. This will save you a lot of time in the future!

## Prizes, grants, distinctions, and scholarships

There are large numbers of these available at medical school both internally and externally, seek these out and submit for them. Also, make sure you get as many prizes and awards for your intercalated BSc (if you choose to do one) and your elective. As a doctor, there are also prizes available, as well as research grants and fellowships.

### Top tip

Not as many people apply for essay prizes as you’d think.

## Displaying leadership and teamwork

At medical school this is manifest as being President of your Surgical Society, or being involved with your Students Union. As a doctor, points are given for being a foundation year representative or being a Mess President. Being effective in positions of responsibility requires you to know how to manage your time, be able to communicate effectively, and to get results through the responsible and efficient delegating of tasks.

### Top tip

This doesn’t have to be medically based, for example, Lead of a charity.

## Teaching experience

Throughout your training as a clinician, you will not only be learning yourself but you will be teaching those less experienced. Thus, teaching skills and experience are viewed favorably. As a medical student, getting involved with peer tutoring of lower years is good experience. As a doctor, designing, organizing, and carrying out regular, regional teaching with feedback is much more impressive than ad-hoc teaching.

### Top tip

Ensure you keep feedback forms as evidence of your teaching and an attendee list.

## Commitment to specialty

Clear examples of your commitment to that specialty need to be demonstrated. This can be in the form of electives, audits, student-selected modules, qualifications (eg, Membership to the Royal College of Surgeons MRCS), or membership of a society.

### Top tip

These can be started early in medical school, the longer the commitment, the more impressive it is.

## Courses

To show commitment to specialty, it is beneficial to have done courses relevant to your field. Courses relevant to surgery include Basic Surgical Skills Course (BSS), Care of the Critically Ill Surgical Patient (CCrISP), Systematic Training in Acute Illness Recognition and Treatment for Surgery (START), Advanced Trauma Life Support (ATLS). Many of the medical specialities also hold careers days for students or trainees interested in a particular speciality, for example annual DermSchool for those interested in dermatology. These courses are often expensive and add up both in time and money. Therefore, take full use of any study budget and study leave allocated to you. Furthermore, be aware that fully certified courses are also run in less central locations and can be much cheaper if you are willing to travel.

### Top tip

Keep your certificates as proof of attendance and completion.

## Clinical/procedural

Keeping a record of which procedures you’ve seen and done shows your competency and commitment. As a student or doctor, be keen to observe and learn new skills, especially in your field of interest.

### Top tip

Keep a log book of what you’ve seen/done, with locations and dates

## Nonmedical experience and qualifications

These can demonstrate that you are a well-rounded individual with a broad view of life, as well as an appreciation of what learning new concepts and types of knowledge can do for your efficiency. You should also demonstrate your personality and individuality by maintaining involvement with extracurricular activities that interest you (everyone wants to work with an interesting and charismatic person).

### Top tip

You’re more than just a doctor/medical student—show it!

## Summary

Ultimately, doing your best to get shortlisted using the advice above will end up making you a better doctor. That is exactly why this forms the criteria for shortlisting—those who have been successful in these areas make well-rounded and efficient clinicians who are able to deal with a broad array of challenges, situations, and professional commitments.

